# TNA‐Mediated Antisense Strategy to Knockdown Akt Genes for Triple‐Negative Breast Cancer Therapy

**DOI:** 10.1002/smtd.202400291

**Published:** 2024-05-23

**Authors:** Pan Li, Shixue Zheng, Hoi Man Leung, Ling Sum Liu, Tristan Juin Han Chang, Alishba Maryam, Fei Wang, Y. Rebecca Chin, Pik Kwan Lo

**Affiliations:** ^1^ Department of Chemistry and State Key Laboratory of Marine Pollution City University of Hong Kong Tat Chee Avenue Kowloon Hong Kong SAR P. R. China; ^2^ Tung Biomedical Sciences Centre Department of Biomedical Sciences City University of Hong Kong Tat Chee Avenue Kowloon Hong Kong SAR P. R. China; ^3^ Department of Chemistry Molecular Sciences Research Hub Imperial College London White City Campus Wood Lane London W12 0BZ U.K.; ^4^ The Tenth Affiliated Hospital Southern Medical University (Dongguan People's Hospital) Dongguan 523059 P. R. China; ^5^ Key Laboratory of Biochip Technology Biotech and Health Care Shenzhen Research Institute of City University of Hong Kong Shenzhen 518057 P. R. China

**Keywords:** AKT, antisense oligonucleotides, gene silencing, threose nucleic acid, triple‐negative breast cancer

## Abstract

Triple‐negative breast cancer (TNBC) remains a significant challenge in terms of treatment, with limited efficacy of chemotherapy due to side effects and acquired drug resistance. In this study, a threose nucleic acid (TNA)‐mediated antisense approach is employed to target therapeutic Akt genes for TNBC therapy. Specifically, two new TNA strands (anti‐Akt2 and anti‐Akt3) are designed and synthesized that specifically target Akt2 and Akt3 mRNAs. These TNAs exhibit exceptional enzymatic resistance, high specificity, enhance binding affinity with their target RNA molecules, and improve cellular uptake efficiency compared to natural nucleic acids. In both 2D and 3D TNBC cell models, the TNAs effectively inhibit the expression of their target mRNA and protein, surpassing the effects of scrambled TNAs. Moreover, when administered to TNBC‐bearing animals in combination with lipid nanoparticles, the targeted anti‐Akt TNAs lead to reduced tumor sizes and decreased target protein expression compared to control groups. Silencing the corresponding Akt genes also promotes apoptotic responses in TNBC and suppresses tumor cell proliferation in vivo. This study introduces a novel approach to TNBC therapy utilizing TNA polymers as antisense materials. Compared to conventional miRNA‐ and siRNA‐based treatments, the TNA system holds promise as a cost‐effective and scalable platform for TNBC treatment, owing to its remarkable enzymatic resistance, inexpensive synthetic reagents, and simple production procedures. It is anticipated that this TNA‐based polymeric system, which targets anti‐apoptotic proteins involved in breast tumor development and progression, can represent a significant advancement in the clinical development of effective antisense materials for TNBC, a cancer type that lacks effective targeted therapy.

## Graphical abstract

A TNA‐mediated antisense approach selectively targets and suppresses Akt2 and Akt3 genes, providing a promising therapeutic strategy for TNBC. The designed TNAs convincingly inhibit tumor growth. The TNA system shows great potential as a cost‐effective and scalable platform for TNBC treatment due to its remarkable resistance to enzymatic degradation, use of inexpensive synthetic reagents, and simple production procedures.

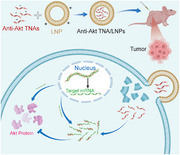

## 1 Introduction

Breast cancer is the most prevalent malignancy in women worldwide.^[^ ^1^ ^]^ Triple‐negative breast cancer (TNBC), a highly aggressive subtype of breast cancer, accounts for ≈15–20% of breast cancers. It is immunohistochemically characterized by a lack of expression of estrogen receptor (ER), progesterone receptor (PR), and human epidermal growth factor receptor 2 (HER2).^[^ ^2^ ^]^ This malignancy is associated with poor prognosis, resulting in low recovery rates and survival rates for TNBC patients. Survival for TNBC patients is significantly shorter compared to other types of breast cancer, with a mortality rate of 40% within 5 years of diagnosis and 75% within 3 months of recurrence.^[^ ^3^ ^]^ TNBC does not respond to hormonal therapy or targeted molecular therapy due to its specific molecular phenotype.^[^ ^4^ ^]^ Currently, the standard treatment for TNBC patients involves surgical excision followed by systemic chemotherapy. However, the majority of TNBC patients still face a high risk of relapse and mortality because of the limited efficacy of chemotherapy, which is hindered by severe side effects, poor treatment response, and acquired drug resistance.^[^ ^5^, ^6^ ^]^ Consequently, there is an urgent need for new treatment modalities to manage.

The use of nucleic acid‐based antisense technology to regulate the target gene expression in TNBC therapy is emerging.^[^ ^7^ ^]^ The desired sequence of MicroRNAs (miRNAs) and small interfering RNAs (siRNAs) are designed to target specific genes such as PDCD4 and HoxD10,^[^ ^8^ ^]^ EGFR, BRD4,^[^ ^9^ ^]^ TGFβ1,^[^ ^10^ ^]^ Notch 1,^[^ ^11^ ^]^ PKM2,^[^ ^12^ ^]^ and survivin,^[^ ^13^ ^]^ thereby disrupting signaling pathways associated with TNBC.^[^ ^14^, ^15^ ^]^ The synergistic effects of combined chemotherapeutic agents and RNAi therapy for TNBC tumor suppression have been widely observed. For example, Deng et al. revealed that combining doxorubicin (DOX) with miRNA‐34a not only significantly boosted the anti‐tumor activity of DOX by inhibiting the expression of anti‐apoptotic protein Bcl‐2 in MDA‐MB‐231 cells but also suppressed the migration of TNBC cells by regulating Notch 1 signaling.^[^ ^16^ ^]^ Additionally, a recent study reported that the combined treatment of TGFβ1 siRNA and DOX reduced cell proliferation and mitochondrial activity, leading to apoptosis activation in Hs578T cells and enhancing the therapeutic effects of DOX.^[^ ^10^ ^]^ Unfortunately, the use of natural nucleic acids for gene therapy faces several challenges, including the in vivo systemic administration of free natural DNA/RNAs. Natural nucleic acids are highly susceptible to degradation by serum nucleases and extracellular enzymes and are rapidly deactivated through processes such as cell surface recycling, lysosomal degradation within the cytosol, and release from intracellular compartments upon introduction into cells or animals. These factors collectively make the systemic delivery of natural RNAs challenging in gene therapy approaches.^[^ ^17^, ^18^, ^19^ ^]^

Inspired by recent advancements and breakthroughs in gene therapy, antisense oligonucleotides (ASOs), which are chemically modified nucleic acids, have emerged as potential alternatives for the treatment of TNBC. To enhance their properties, chemical modifications can be introduced to the nucleobases, phosphodiester backbone, and/or sugar moieties of ASOs. These modifications result in improved resistance to nucleases, favorable pharmacokinetic properties, increased stability in duplex‐forming, and reduced toxicity in cells and animals.^[^ ^20^ ^]^ For example, a menin‐ASO with phosphorothioate (PS) backbone has been designed to specifically bind to menin mRNA, leading to the inhibition of cell proliferation and enhanced apoptosis in Hs578T cells. In vivo studies have demonstrated significant suppression of TNBC progression using this PS‐ASO.^[^ ^21^ ^]^ Similarly, eight PS‐ASOs targeting the long non‐coding RNA TROJAN have also shown substantial suppression of TNBC progression.^[^ ^22^ ^]^ Another study by Hu et al. utilized locked nucleic acids (LNAs) that target LINK‐A, an oncogenic lncRNA, to reverse the downregulation of antigen presentation and intrinsic tumor suppression. They demonstrated the stabilization of the antigen peptide‐loading complex (PLC) components, Rb and p53 while sensitizing mammary gland tumors to immune checkpoint blockers. This approach effectively enhanced the effectiveness of immunotherapy in TNBC treatment.^[^ ^23^ ^]^ Nevertheless, the use of unnatural PS‐ASO and LNA does have certain limitations. These include suboptimal cellular uptake, a tendency for self‐annealing, relatively weak binding affinity to natural DNA/RNAs, the potential for eliciting an immune response, and elevated toxicity when employed in disease treatment.^[^ ^24^ ^]^

Threose nucleic acid (TNA), an RNA‐like polymer, consists of a backbone comprised of a four‐carbon threose sugar group with phosphodiester linkages occurring at the 2’‐ and 3’‐vicinal positions of the sugar ring.^[^ ^25^ ^]^ In comparison to natural nucleic acids, sequence‐controlled TNA polymers show stronger specificity and affinity toward their complementary RNAs. These synthetic TNA polymers have demonstrated cellular internalization in various cell lines, including HeLa, MCF‐7, HepG2, HEK293, and A549.^[^ ^26^ ^]^ Moreover, synthetic TNA polymers have exhibited high stability in extreme pH conditions, human blood serum, fetal bovine serum, and when stored in various buffer solutions at room temperature for up to six months, highlighting their bio‐stability. As functional biomaterials, TNAs can be safely administered into animal models via tail vein injection, with no induction of pathological changes or acute functional and structural damage to the renal systems.^[^ ^27^ ^]^ In recent years, we have reported a cost‐efficient strategy for synthesizing TNA phosphoramidite and used it for the solid‐phase synthesis of sequence‐designed TNAs, achieving high synthetic yield.^[^ ^26^ ^]^ Recent studies have shown the potential of sequence‐designed TNAs as effective antisense oligonucleotides or XNAzymes for suppressing specific gene expression in common cancerous cells, showing antitumor activity without adverse toxicity.^[^ ^28^, ^29^, ^30^, ^31^, ^32^, ^33^ ^]^

Herein, we have designed and synthesized two novel TNA strands, namely anti‐Akt2, and anti‐Akt3, which specifically target the corresponding Akt2 and Akt3 mRNAs. These TNAs were used to investigate their potential antitumor activity in TNBC cells and triple‐negative breast carcinoma xenograft models. The AKT gene plays a pivotal role in the PI3K/AKT/mTOR signaling pathway. Dysregulation of this pathway, commonly observed in TNBC, contributes to enhanced cancer cell survival, growth, and chemotherapy resistance.^[^ ^34^ ^]^ The three isoforms of Akt (Akt1, Akt2, and Akt3) are encoded by distinct genes, exhibit high sequence similarity, and are activated through near‐identical mechanisms.^[^ ^35^ ^]^ However, they demonstrate different or even opposing functions in regulation of TNBC development.^[^ ^36^, ^37^ ^]^ Previous studies confirmed that targeting the AKT pathway could be an effective therapeutic strategy for TNBC. For example, Chin et al. reported that shRNA‐mediated knockdown of Akt3 expression significantly inhibits the growth of MDA‐MB‐231 or MCF10DCIS cells in 3D cultures and in tumor‐treated animal models, in comparison to shRNA suppressing of Akt1 and Akt2 expression.^[^ ^38^ ^]^ In contrast, PTEN‐deficient TNBC spheroids are also dependent on Akt2‐mediated survival signaling.^[^ ^39^ ^]^ Interestingly, although Akt1 promotes tumor induction, it has been shown to inhibit migration, invasion, and metastasis of breast cancer cells.^[^ ^40^, ^41^ ^]^ Therefore, depleting Akt1 may have undesired effects of enhancing metastatic dissemination.

**Scheme 1:**
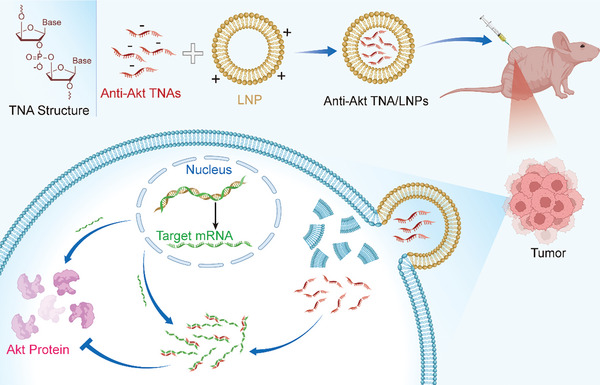
Schematic representation of the working principle for the anti‐Akt TNAs.

In this study, we synthesized sequence‐designed anti‐Akt TNA polymers using well‐established cyanoethylphosphoramidite chemistry. These TNAs demonstrated remarkable enzymatic resistance, high specificity, enhanced binding affinity with their target RNA molecules, and improved cellular uptake efficiency in various TNBC cell lines, surpassing the performance of natural nucleic acids. The results showed significant inhibition of the target mRNA and protein expressions in both 2D and 3D TNBC cells, in comparison to cells treated with scrambled TNAs. To explore their antitumor properties, the anti‐Akt TNAs were formulated with lipid nanoparticles for subcutaneous injection. Treatment of TNBC‐bearing animals with these targeted TNA polymers resulted in reduced tumor sizes and decreased expression of the target protein, as compared to control groups. The silencing of the corresponding Akt genes using these highly biostable TNAs facilitated a potent apoptotic response in TNBC and suppressed tumor cell proliferation (**Scheme** **1**).

## 2 Results and Discussion

### 2.1 Design, Synthesis, and Characterization of Sequence‐Specific TNA Polymers

In this study, we designed TNA‐based antisense oligonucleotides, specifically anti‐Akt2 TNA and anti‐Akt3 TNA, to partially complement the sequence of target Akt2 and Akt3 mRNAs respectively. As a control, a scramble TNA strand with the same number of nucleotides but in random sequences was also designed. These sequence‐designed TNA oligonucleotides were synthesized using an automatic nucleic acid synthesizer, following our modified solid‐phase synthetic prot^oc^ols involving cyanoethylphosphoramidite chemistry.^[^ ^26^ ^]^ After purification through PAGE, pure sequence‐designed TNA strands were obtained and further characterized using high‐resolution matrix‐assisted laser desorption ionization time‐of‐flight (MALDI‐TOF) mass spectrometry studies (Figure S1, Supporting Information). The sequences of the TNAs and DNA/RNA oligonucleotides used in this project are listed in Table S1, (Supporting Information). Additionally, Cy3 fluorophore was employed to label the two antisense oligonucleotides (Cy3‐anti‐Akt2 TNA and Cy3‐anti‐Akt3 TNA) for cellular imaging purposes. The high coupling efficiency of the four TNA monomers and the high production yield of the TNA polymers provide strong evidence for the feasibility of sequence‐independent synthesis of TNAs.^[^ ^26^ ^]^

### 2.2 The Binding Specificity and Affinity of Antisense Akt TNAs toward their Targets

**Figure 1:**
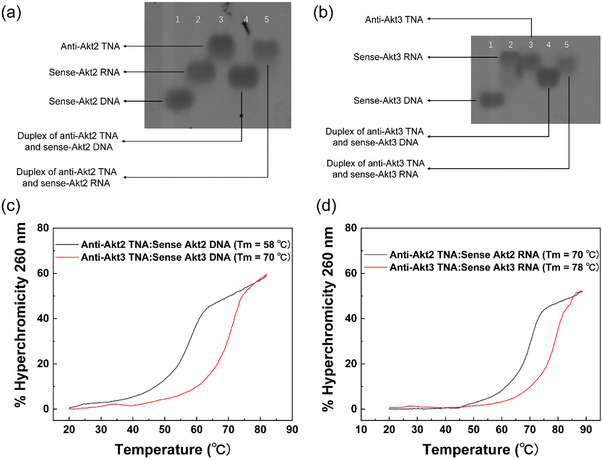
Native PAGE analyses of the bind specificity of the a) anti‐Akt2 TNA and b) anti‐Akt3 TNA, with their complementary DNA and/or RNA. c) Thermal denaturation studies of (c) anti‐Akt2 TNAs and d) anti‐Akt3 TNAs with their complementary DNA/RNA strands.

According to its strand orientation, specificity, and pairing strength, TNA is considered a potential progenitor of RNA and exhibits similar nucleobase pairing capability to natural RNA.^[^ ^42^ ^]^ Native PAGE analyses were performed to investigate the binding specificity of anti‐Akt2 TNA and antiAkt3 TNA to sense‐Akt2 and ‐Akt3 oligonucleotides respectively. As shown in **Figure** **1**a,b, unique product bands (lanes 4 and 5) with higher mobility compared to TNA (lane 3) alone were observed, along with the disappearance of the complementary partners, upon mixing either anti‐Akt2 TNA or anti‐Akt3 TNA with the corresponding complementary sense‐DNA (lane 1) or ‐RNA (lane 2). This unexpected gel mobility of the higher molecular size of the duplex products is attributed to the secondary structure formation of anti‐Akt TNAs, which adopt highly ordered conformations with lower mobility than regular single‐stranded oligonucleotides. The secondary structure of anti‐Akt TNAs gets disrupted when they recognize and bind to their complementary oligonucleotides, resulting in faster mobility than TNA alone. As controls, anti‐Akt TNAs did not interact with the non‐complementary oligonucleotides (Figure S2, Supporting Information). Thermal denaturation analyses revealed that anit‐Akt TNAs exhibit stable base pairing and improved binding affinity toward their complementary RNAs compared to their complementary DNAs. As shown in Figure 1c, the melting temperature (*T*_m_) values of anti‐Akt2 TNA:sense‐Akt2 RNA or anti‐Akt3 TNA:sense‐Akt3 RNA complexes were ≈70 °C and 78 °C, respectively, which were ≈12 °C and 8 °C higher than those for the anti‐Akt2 TNA:sense‐Akt2 DNA or anti‐Akt3 TNA:sense‐Akt3 DNA complexes, respectively. Additionally, our group previously reported that TNA‐based polymers showed higher *T*_m_ values compared to phosphorothioate‐modified DNAs.^[^ ^26^ ^]^ Compared to commonly used natural nucleic acid‐based antisense agents, our PAGE and thermal denaturation analyses confirmed the high specificity and enhanced binding affinity of the sequence‐designed anti‐Akt TNA with their corresponding target RNA molecule. These findings are potentially significant and beneficial for effectively silencing the Akt gene in TNBC therapy.

### 2.3 Biological Properties of Sequence‐Designed Anti‐Akt TNAs

**Figure 2:**
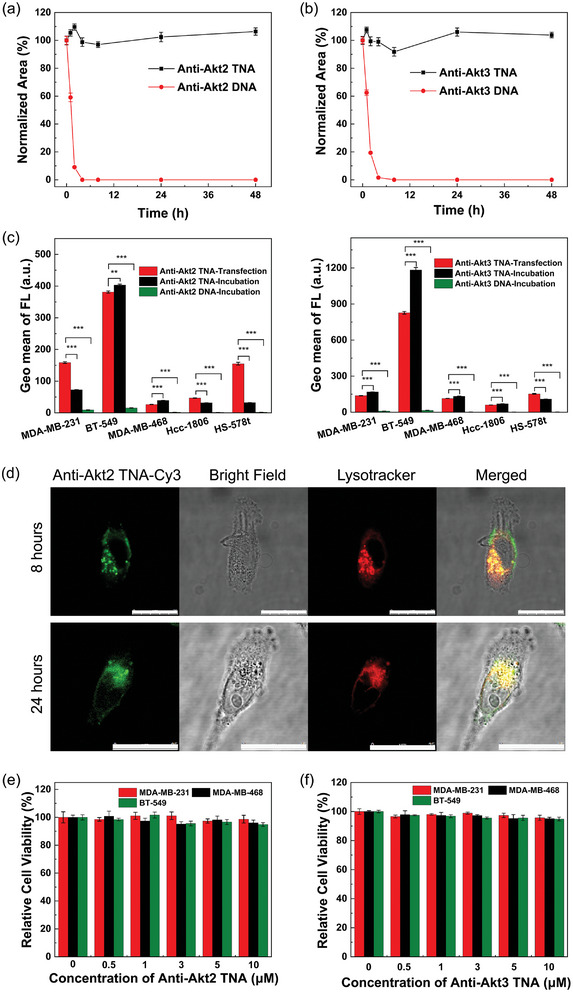
Quantitative analyses of enzymatic degradation of a) anti‐Akt TNA/DNA and b) anti‐Akt TNA/DNA under 10% fetal bovine serum (FBS) as a function of incubation time. c) Flow cytometry analysis of the uptake efficiency of anti‐Akt TNAs (in blue and red colors) vs anti‐Akt DNAs (in green color) in various TNBC cell lines; d) Colocalized confocal fluorescence images of anti‐Akt2 TNA‐treated BT‐549 cells after incubating for 8 and 24 h; MTT tests of e) anti‐Akt2 TNA and f) anti‐Akt3 TNA in different cell lines including BT‐549, MDA‐MB‐231 and MDA‐MB‐468 cells, the antisense TNA was incubated with the cells, which was not transfected into the cells.

The biomedical application of natural DNA or RNA is often hindered by their short half‐lives, typically around or <30 min in biological conditions.^[^ ^43^ ^]^ To evaluate the biostability of TNA, serum stability assays were conducted to investigate its resistance to enzymatic degradation compared to natural DNAs. TNA and DNA samples were incubated with 10% fetal bovine serum (FBS) for varying incubation times: 1, 2, 4, 8, 24, and 48 h. Denaturing PAGE analyses were employed to determine and compare the degree of degradation between TNAs and DNAs (Figure S3, Supporting Information). The normalized band intensity, as shown in **Figure** **2**a,b, revealed that the half‐life (*t*_1/2_) of anti‐Akt2 DNA and anti‐Akt3 DNA were ≈1.5 and 2.3 h, respectively, with complete degradation occurring after 2 and 4 h, respectively. In contrast, anti‐Akt2 TNA and anti‐Akt3 TNA remained intact without degradation even after 48 h of incubation. The enhanced biological stability observed in TNA, in comparison to natural oligonucleotides, can be attributed to its distinctive structural features. Firstly, TNA incorporates a synthetic threose sugar composed of four carbon atoms, deviating from the natural ribose sugar found in DNA, which consists of five carbon atoms. Despite this modification, TNA retains the same nucleobases and phosphodiester bonds as DNA. Secondly, TNA lacks the 2'‐hydroxyl group present in the threose sugar backbone. As a result, TNA does not provide recognition sites for nucleases, resulting in significantly stronger resistance against degradation. This limited enzyme recognition contributes to the higher enzymatic resistance observed for TNA in serum tests, making it a more reliable choice for long‐term studies in biological environments after administration. However, it should be noted that TNA may still degrade after certain periods of time, addressing the concerns related to excretion and accumulation.

To compare the cellular efficiency of natural nucleic acids and TNAs, flow cytometry studies were conducted on five different types of TNBC cells. These cells were subjected to three conditions: incubation with natural DNAs, incubation with TNAs, and transfection of TNAs. The intracellular uptake efficiency of anti‐Akt TNAs varied depending on the cell type. When incubated overnight with TNBC cell lines such as MDA‐MB‐231, MDA‐MB‐468, BT‐549, HCC1806, and Hs578T cells, the overall cellular uptake of anti‐Akt TNAs was significantly higher than that of natural nucleic acids (Figure 2c). However, colocalized confocal fluorescence imaging of anti‐Akt TNA‐treated TNBC cells revealed that only a small amount of anti‐Akt TNAs were localized in the cytoplasm after 24 h of incubation in MDA‐MB‐231 and BT‐549 cells (Figure 2d; Figure S4, Supporting Information). The majority of the anti‐Akt TNAs remained trapped inside the lysosomes. MTT results also supported this observation, as they showed very low cytotoxicity to the TNBC cells without transfection treatment. The TNA‐treated TNBC cells remained healthy, with a relative cell viability of ≈98% even after incubation at a high TNA concentration of 10 µM for 48 h (Figure 2d,e). Under transfection treatment, we observed successful release of the anti‐Akt TNAs from the lysosomes (Figure S5, Supporting Information). These TNAs predominantly localized in the cytoplasm of TNBC cells, facilitating their action by degrading targeted Akt mRNAs in the cytoplasm. This process effectively silenced the genes, highlighting the potential of the highly biostable TNAs as an alternative therapeutic option for gene silencing applications in TNBC therapy. The aim of this study is to demonstrate the potential of highly biostable TNAs as an alternative therapeutic option for TNBC treatment Consequently, to assess the functional effects of TNA‐mediated therapy in TNBC, appropriate transfection conditions were employed for both 2D and 3D cellular studies using lipofectamine, while lipid nanoparticles were utilized for animal studies.

### 2.4 In Vitro Gene Silencing by Anti‐Akt TNAs in TNBC Cells

**Figure 3:**
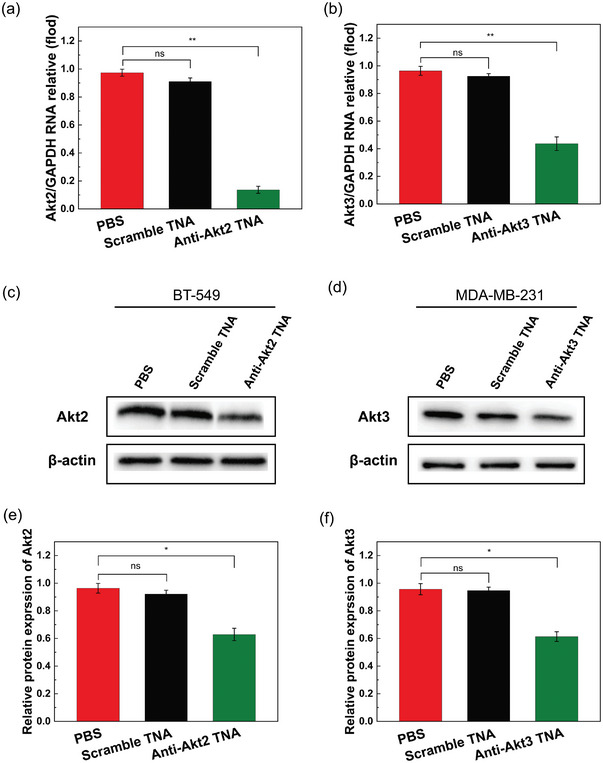
Target mRNA and protein expressions in TNBC cells after treatment with PBS, transfected scramble TNA, and transfected anti‐Akt TNAs. The relative a) Akt2 mRNA level in BT‐549 cells and b) Akt3 mRNA level in MDA‐MB‐231 cells after different treatments. c) Akt2 protein expression level of BT 549 cells and d) Akt3 protein expression level of MDA‐MB‐231 cells after different treatments. Quantitative analyses of e) Akt2 protein and (f) Akt3 protein levels in (c) and (d) respectively. Data are shown as the mean ± standard deviation (SD) (*n* = 3). Error bars, mean ± SD of three independent experiments. *p < 0.05; **p < 0.01. “ns” stands for “not significant,” indicating that the observed differences between the compared groups did not reach statistical significance based on our chosen threshold (*p* > 0.05).

Based on the previous validation that TNAs can form stable double‐stranded structures with their complementary DNA or RNA strands through base pairing, we hypothesized that antisense TNAs can bind to their complementary messenger RNA (mRNA) and inhibit the transfer of genetic information from mRNA to proteins, thereby suppressing target gene expression. To test this hypothesis, we selected Akt2 and Akt3 as target genes to evaluate the gene‐silencing efficacy of the sequence‐designed anti‐Akt TNAs. Firstly, we transfected the anti‐Akt TNAs into various 2D TNBC cells, including BT‐549, MDA‐MB‐468, and/or MDA‐MB‐231 cells, and cultured them at 37 °C for 24 h. Quantitative reverse transcription polymerase chain reaction (RT‐qPCR) analysis indicated that anti‐Akt2 TNA significantly reduced Akt2 mRNA expression by ≈70% in BT‐549 cells (Figure 3a), while anti‐Akt3 TNA inhibited over 40% of Akt3 mRNA expression in MDA‐MB‐231 cells (Figure 3b). In contrast, TNBC cells transfected with scramble TNA did not show any apparent effect, further confirming the high selectivity and specificity of sequence‐specific TNAs in cell‐based studies. At the protein level, western blot (WB) analyses were conducted to validate the silencing efficiency of the target Akt2 and Akt3 genes. Compared to the control group treated with PBS alone, the expression level of target proteins exhibited a reduction of approximately 45% for anti‐Akt2 TNA (Figure 3c,e) and ≈40% for anti‐Akt3 TNA (Figure 3d,f). As the inhibition effect was found to be cell‐type dependent, we did not observe any promising WB result of anti‐Akt2 TNAs in MDA‐MB‐231 and MDA‐MB‐468 cells (Figure S6, Supporting Information). Collectively, these RT‐qPCR and WB results clearly confirmed the feasibility of sequence‐designed anti‐Akt TNAs effectively silencing the corresponding target gene expression at both mRNA and protein levels. Antisense oligonucleotides play a significant role in modulating gene expression within cells through mechanisms such as mRNA cleavage, steric hindrance, or splice switching. In the case of TNA, it was not digested by enzymes, even though DNase I was added during RNA extraction to eliminate DNA contamination. Therefore, the antisense mechanism of TNA is highly independent of RNase H, leading us to believe that the observed decrease in target mRNA and protein levels is achieved by inhibiting or blocking the translation process, followed by inhibition of cDNA synthesis. Our observation is also in good agreement with the RNase H‐mediated digestion studies done by Yu's group.^[^ ^43^ ^]^

### 2.5 Akt Depletion Inhibiting Triple‐Negative Breast Tumor Spheroid Growth by Anti‐Akt TNAs

**Figure 4:**
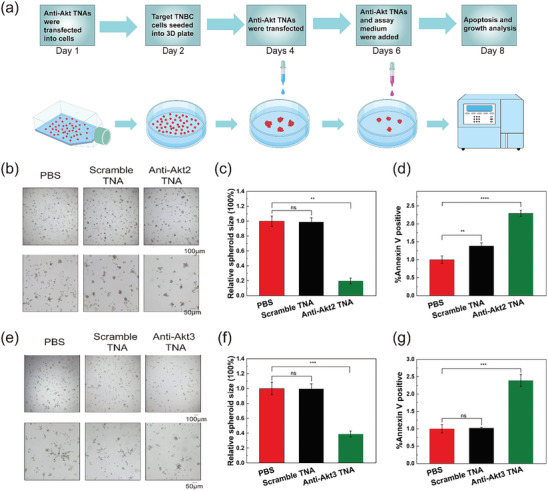
In vitro characterization of Akt isoform in TNBC cells after treatment with PBS, transfected scramble TNA, or transfected anti‐Akt TNAs. a) Timeline illustrating the development of triple‐negative breast tumor spheroids for growth and apoptosis analyses. b) Representative pictures of BT‐549 tumor spheroids. c) The relative sizes of triple‐negative breast tumor spheroids in (b). d) Percentage of annexin V‐positive BT‐549 cells with different treatments. e) Representative pictures of MDA‐MB‐231 tumor spheroids. f) The relative sizes of triple‐negative breast tumor spheroids in (e). g) Percentage of annexin V‐positive MDA‐MB‐231 cells with different treatments. Error bars, mean ± SEM of three independent experiments. *p < 0.05; **p < 0.01; ***p < 0.001, ****p < 0.0001. “ns” stands for “not significant”.

To functionally determine the effect of TNA‐targeted therapy in TNBC cells, a t3D cell culture model was used to investigate cell apoptosis and cell growth in TNBC cells. The 3D cell culture allows cells to grow and interact with the surrounding extracellular framework in three dimensions, mimicking tissues and in vivo tumor environment.^[^ ^44^ ^]^ Following the culture protocols for triple‐negative breast tumor spheroid shown in **Figure** **4**a, we examined the consequences of TNA‐based Akt targeted therapy on tumor spheroid growth and apoptosis using phase‐contrast imaging and flow cytometry, respectively. In our study, we initially performed the first transfection of TNA ASOs in a 2D cell culture system to enhance the uptake efficiency of TNA by the target cells. This initial transfection aimed to ensure a significant amount of TNA was delivered into the cells, setting the stage for subsequent therapeutic effects. On the fourth day following the initial transfection, we conducted a second transfection. By this time, a portion of the TNA from the first transfection would have undergone metabolic processes within the cells. To maintain a sustained drug effect and ensure a sufficient concentration of TNA within the cells, a second transfection was deemed necessary. For the purposes of apoptosis and growth analysis, there was a four‐day duration from the last transfection. To ensure an optimal drug concentration and maximize therapeutic efficacy, we performed a third addition of TNA ASOs. This additional administration of the medication aimed to maintain a consistent drug concentration throughout the experimental duration, thus ensuring the desired therapeutic effect of the TNA ASOs. Compared to PBS‐ and scramble TNA‐treated groups, the bright field images of anti‐Akt TNA‐treated cells exhibited significantly smaller spheroids. Statistically, depletion of Akt2 and Akt3 in anti‐Akt2 TNA‐treated BT‐549 and anti‐Akt3 TNA‐treated MDA‐MB‐231 cells potently inhibited spheroid growth, resulting in a 79.13% and 60.41% reduction in spheroid size, respectively (Figure 4b,c). Furthermore, we assessed the effect of anti‐Akt2 TNA and anti‐Akt3 TNA on cell apoptosis by staining 3D TNBC cells with Annexin V, a marker for cell apoptosis. The percentage of Annexin V‐positive cells was significantly higher in anti‐Akt2 TNA‐treated BT‐549 spheroids and anti‐Akt3 TNA‐treated MDA‐MB‐231 spheroids compared to PBS‐ or scramble TNA‐treated group (Figure 4d). These findings on the growth of 3D spheroids align with previous reports, demonstrating a remarkably selective silencing of Akt2 in BT‐549 3D tumor spheroids and Akt3 in MDA‐MB‐231 3D tumor spheroids, resulting in the disintegration of the spheroids. Similar effects were observed when combining the corresponding Tet‐on Akt shRNA and doxycycline treatments.^[^ ^39^ ^]^ Taken together, these data indicate that Akt isoform targeted TNA treatment significantly inhibits TNBC spheroids growth and induces cell apoptosis in vitro.

### 2.6 Antitumor Effect and Gene Silencing in Xenograft Models for TNBC Therapy

To evaluate the antitumor effects of suppressing the AKT gene, we utilized tumor‐bearing animal models established in BALB/c nude mice by injecting TNBC cells subcutaneously. Given the potent effects of anti‐Akt2 TNA and anti‐Akt3 TNA on PTEN‐deficient BT549 and PTEN‐Wild type MDA‐MB‐231 spheroid growth in 3D models, respectively, we investigated the antitumor effects of TNAs in these two TNBC models. In this study, we utilized EntransterTM transfection reagents (LNP) mixed with anti‐Akt TNAs to form complexes, enabling effective entry into cell tissues. LNP possesses distinctive characteristics, such as strong nucleic acid binding and protection abilities, low toxicity, and minimal induction of immune responses. These features have made LNP widely employed in scientific research, demonstrating excellent therapeutic effects.^[^ ^45^ ^]^ Specifically, LNP consists of nanoscale polymeric materials containing numerous amino functional groups. These amino groups can undergo protonation at physiological pH, enabling them to neutralize the negative charges on the surface of TNA plasmids. This neutralization process leads to the compression of TNA molecules, reducing their volume from an extended structure to smaller particles. Consequently, the transfection reagent safeguards and concentrates nucleic acids, preventing their degradation by bodily fluids and enhancing their entry into cell tissues through physical effects. This process ultimately produces the desired effects. Upon internalization into cells via endocytosis, the transfection complexes form endosomes. Subsequently, TNA is released from the endosomes, enters the cytoplasm, and ultimately reaches the target sites for transcription and expression. So far, the systemic validation of LNP has confirmed its stability and biocompatibility for TNA delivery. For DLS analysis, 40 µL of LNP was mixed with 360 µL of autoclaved water, resulting in a total volume of 400 µL. The LNP particles exhibited two size distributions, ≈7.2 ± 1.7 and 206.3 ± 3.1 nm in diameter (Figure S7a, Supporting Information). Moreover, we observed minimal changes in particle size over a three‐day incubation period, indicating the relative stability of the LNPs over time (Figure S7b, Supporting Information). These findings align with the TEM results reported by Wang et al., where the larger size distribution of LNPs was attributed to agglomeration.^[^ ^46^ ^]^ To evaluate its biocompatibility, an MTT assay was performed to assess the cytotoxicity of LNPs on MDA‐MB‐231 and BT‐549 cells at different concentrations. As shown in Figure S8 (Supporting Information), increasing the LNP concentration resulted in a relatively high cell viability. Even at a high concentration of 20 µg mL^−1^, which was used for in vivo studies, the cell viability remained at 98%. These results indicate the good biostability and biocompatibility of LNP for this TNA delivery system.

**Figure 5:**
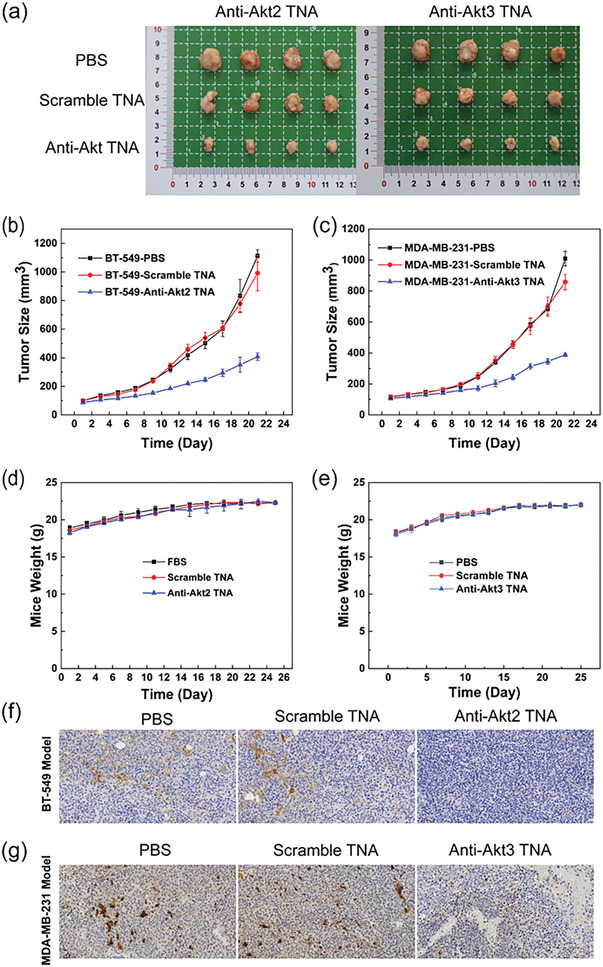
Sequence‐designed anti‐Akt TNAs for in vivo antisense TNBC therapy. a) Photos of harvested tumors on day 21 from each group. Tumor growth curves of the b) BT‐549 and c) MDA‐MB‐231 tumor‐bearing mice (*n* = 4) after i.t. injection of PBS, scramble TNA, and the corresponding anti‐Akt TNAs. Relative body weight change curves of each group of d) BT‐549 and e) MDA‐MB‐231 tumor‐bearing mice during the tumor‐growth inhibition study. Arrows represent the day for injection of samples. Error bars are standard errors of four mice in each group. Representative images of IHC staining of the tumor tissues harvested from f) BT‐549‐bearing mice and g) MDA‐MB‐231‐bearing mice with different treatments.

Once the tumor volume reached ≈100 mm^3^, the mice were randomly assigned to three groups: a control group treated with PBS (control 1), a second control group treated with scramble TNA/LNP (control 2), and an experimental group treated with anti‐Akt TNAs/LNP (experimental group). Each group consisted of four mice. The overall tumor volume, tumor size, and body weight of mice were monitored and measured over time following intratumoral (i.t.) administration of PBS, scramble TNA/LNP, and anti‐Akt2/LNP or anti‐Akt3 TNA/LNP every two days. The mice were carefully monitored for 21 days to assess the in vivo therapeutic efficacy. After 21 days of i.t. injection with the three treatments, the tumors were harvested from the nude mice, and their images are displayed in **Figure** **5**a. Compared to the two control groups, a substantial reduction in triple‐negative breast tumor growth was observed in mice treated with anti‐Akt TNAs over time (Figure 5b,c). In the PBS‐treated group and the scramble TNA‐treated groups, the tumors grew rapidly, with the overall tumor size increasing by ≈8‐ to 10‐fold in BT‐549‐bearing mice with the treatment of anti‐Akt2 TNAs and 10‐ to 11‐fold in MDA‐MB‐231‐bearing mice with the treatment of anti‐Akt3 TNAs. In contrast, the animal groups treated with either anti‐Akt2 TNAs or anti‐Akt3 TNAs showed significant inhibition of tumor growth, resulting in only about a 3‐fold increase in the overall tumor size on day 21. It is important to note that the tumor sizes shown in Figure 5a were obtained by extracting the tumors from mice after 21 days of drug administration. On the other hand, the line graphs in Figure 5b,c depict the measurements of tumor sizes from the first day after drug administration in mice. The disparity in time points introduces inherent variations and potential inaccuracies in the measurements. However, upon closer examination of the line graphs in Figure 5b,c, it can be observed that on the 21st day, both in the BT‐549 animal model and the MDA‐MB‐231 animal model, the tumor sizes in the PBS‐treated group appear slightly larger than those in the Scramble TNA group. This observation is consistent with the findings presented in Figure 5a, where the tumors in the PBS group were indeed larger than those in the Scramble TNA group after 21 days of drug administration. Furthermore, the inclusion of error bars in Figure 5b,c provides a visual representation of the variability in tumor sizes within each group. These error bars support the accuracy and reliability of the measurements presented in Figure 5a. Furthermore, the overall body weights of mice treated with or without TNAs did not show significant differences (Figure 5d,e).

**Figure 6:**
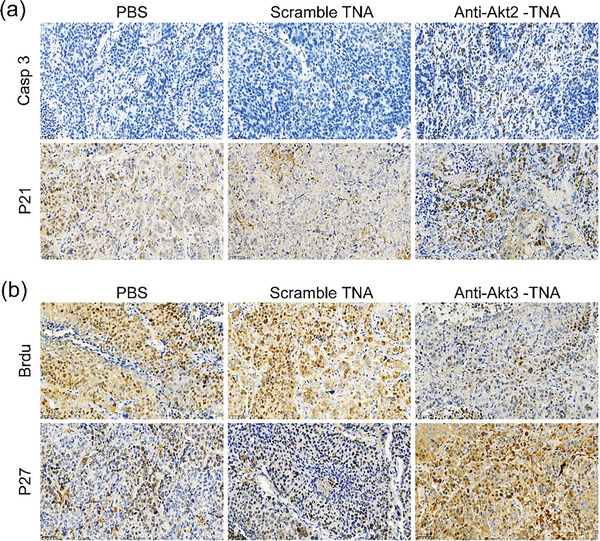
IHC staining of the tumor tissues harvested from the a) BT‐549‐bearing mice and b) MDA‐MB‐231‐bearing mice with different treatments. Tumor tissues were fixed with formaldehyde, embedded in paraffin, sectioned, subjected to antigen retrieval, labeled with specific antibodies, and underwent DAB staining reaction for observation under a microscope and selection of fields of view. Antibodies of AKT2, AKT3, and P27 are purchased from Abcam, and antibodies of P21, Caspase‐3, and BrdU are purchased from CST.

To validate the efficacy of silencing the target Akt genes in TNBC therapy, we performed immunohistochemistry (IHC) on xenografts harvested on day 21. In the IHC staining, the dark brown color represents the presence of the target protein, regardless of the specific target protein being examined. The blue color, on the other hand, represents the staining of cell nuclei. In Figure 5f,g, which shows extracted tumor tissue from the BT‐549 cell model and MDA‐MB‐231 cell model, the dark brown color observed in the PBS group and scramble TNA group represents the target Akt2 and Akt3 proteins. In the anti‐Akt2/3 TNA treated group, a significant reduction in the dark brown color is observed, indicating a notable inhibition and significant decrease in the expression level of the target proteins. Previous studies have shown that the inhibition of Akt2 leads to regression of prostate xenografts, accompanied by caspase‐3 activation and induction of p21.^[^ ^39^ ^]^ Akt isoforms have also been implicated in regulation of p27 expression at both the transcriptional and posttranslational levels.^[^ ^47^, ^48^ ^]^ In **Figure** **6**a, the brown color of Casp3 staining indicates the presence of the apoptotic protein, serving as an indicator of cell apoptosis. Thus, in the PBS and Scramble TNA groups, minimal or almost no brown color is observed, suggesting low levels of Casp3 protein and a rare occurrence of cell apoptosis. However, in the anti‐Akt2 TNA group, the brown color intensifies, indicating a significant increase in Casp3 protein and a notable enhancement of cell apoptosis. Additionally, the silencing of Akt2 in BT‐549‐bearing animals resulted in an upregulation of p21 expression, suggesting p21 as a downstream effector of Akt2 in the apoptotic response to anti‐Akt2 TNA treatment (Figure 6a). On the other hand, IHC staining of bromodeoxyuridine (BrdU) and p27 was performed to assess the knockdown effect of Akt3 in MDA‐MB‐231‐bearing animals on cell proliferation and p27 expression. In Figure 6b, the brown color represents the staining result of BrdU, a synthetic analog of thymidine, which serves as an indicator of cell proliferation. In the PBS and Scramble TNA groups, most cells display a deep brown color, indicative of a normal level of Brdu and active cell proliferation. However, in the anti‐Akt3 TNA treatment group, the intensity of the dark brown color is significantly reduced, suggesting a noticeable decrease in Brdu level and implying a substantial inhibition of cell proliferation. This further resulted in an increase in p27 expression, indicating that the silencing of Akt3 by anti‐Akt3 TNAs effectively modulates tumor growth in TNBC xenografts by suppressing tumor cell proliferation. These findings are consistent with the effects observed in previous studies using Tet‐on Akt shRNA and doxycycline treatments.^[^ ^38^, ^39^ ^]^ Overall, our study confirms that sequence‐designed anti‐Akt TNAs can effectively target different Akt isoforms in distinct triple‐negative cells, providing a promising approach for TNBC therapy with different therapeutic mechanisms.

### 2.7 In Vivo Safety Evaluation of Anti‐Akt TNAs

**Figure 7:**
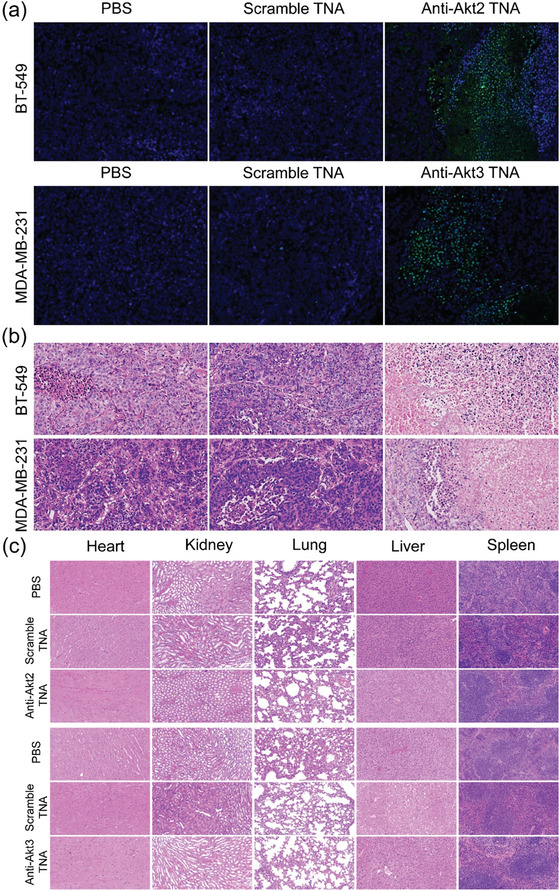
Representative images of a) TUNEL and b) H&E staining of the tumor tissues harvested from BT‐549 tumor‐bearing and MDA‐MB‐231 tumor‐bearing mice with different treatments. c) Histological analysis of major organs such as heart, kidney, lung, liver, and spleen, harvested from BT‐549 tumor‐bearing and MDA‐MB‐231 tumor‐bearing mice on day 21 after different treatments.

We also conducted comprehensive histopathological and histological analyses on tumor‐bearing mice and their major organs to confirm the biosafety of anti‐Akt TNAs in therapeutic applications. In comparison to the two animal control groups, we observed increased levels of apoptosis in both BT‐549 and MDA‐MB‐231 tumor‐bearing mice, as evidenced by the elevated green fluorescence intensity of DNA fragmentation in the terminal deoxyribonucleotidyl transferase‐mediated dUTP nick end labeling (TUNEL) assay (**Figure** **7**a). Histopathological examination using H&E staining further indicated that substantial apoptosis and necrosis of TNBC cells within the BT‐549 and MDA‐MB‐231 tumor tissues in the anti‐Akt2 TNA‐ and anti‐Akt3 TNA‐treated animal groups, as compared to the control groups (Figure 7b). Additionally, we assessed the potential in vivo side effects by histologically analyzing the collected major organs in the three different animal groups. Encouragingly, the H & E staining results showed no detectable lesions or no obvious damage in the heart, liver, spleen, lungs, and kidneys after anti‐Akt TNA treatments in both BT‐549 and MDA‐MB‐231 tumor‐bearing mice (Figure 7c). Taken together, both in vitro and in vivo results provide compelling evidence that anti‐Akt TNA, as an effective antisense therapeutic agent, exhibits excellent biosafety and superior efficacy against TNBC.

## 3 Conclusion

In conclusion, this study demonstrated the potential of a TNA‐mediated antisense approach as a promising therapeutic strategy for TNBC. The designed and synthesized TNA strands, specifically targeting Akt2 and Akt3 mRNAs, exhibited remarkable enzymatic resistance, high specificity, improved binding affinity, and enhanced cellular uptake efficiency compared to natural nucleic acids. In both 2D and 3D TNBC cell models, the TNAs effectively inhibited the expression of their target mRNA and protein, surpassing the effects of scrambled TNAs. Moreover, in TNBC‐bearing animals, the administration of targeted anti‐Akt TNAs in combination with lipid nanoparticles led to reduced tumor sizes, decreased target protein expression, accelerated apoptotic responses and suppressed tumor cell proliferation in vivo. This study introduces a novel approach using TNA polymers as new antisense materials for TNBC therapy. The TNA system offers advantages over conventional miRNA‐ and siRNA‐based treatments, including cost‐effectiveness, scalability, and remarkable enzymatic resistance. The use of inexpensive synthetic reagents and simple production procedures further enhance its potential as a viable platform for TNBC treatment. By targeting anti‐apoptotic proteins involved in breast tumor development and progression, this TNA‐based polymeric system represents a significant advancement in the development of effective antisense materials for TNBC, a cancer type known for its poor response to chemotherapy and radiation treatments. These findings hold promise for the clinical development of TNBC therapies tailored toward improving patient outcomes.

## 4 Experimental Section

### Reagents and Materials

Sephadex G‐25 (super fine DNA grade) was used as purchased from Amersham Biosciences. Fetal bovine serum (FBS), phosphate‐buffered saline (PBS), Dulbecco's modified Eagle medium (DMEM), penicillin‐streptomycin solution, trypsin, and organelle trackers/markers were purchased from Invitrogen. 1 × TAMg buffer was composed of 45 mm Tris and 7.6 mm MgCl_2_, with pH adjusted to 7.8 using glacial acid. 1 × TBE buffer was composed of 90 mm Tris and boric acid and 1.1 mm EDTA, with a pH of ≈8.2. All reagents were of reagent‐grade quality and used as received from J&K (China) unless otherwise indicated. Anhydrous dichloromethane (DCM) was distilled over CaH_2_. All other solvents were of technical grade unless noted. l‐Ascorbic acid, calcium carbonate, anhydrous oxalic acid, para‐toluenesulfonic acid monohydrate, benzoyl chloride, 4‐dimethylaminopyridine, imidazole, tert‐butyldiphenylchlorosilane, adenine, thymine, N4‐benzoylcytosine, N2,9‐diacetylguanine, diphenylcarbamic chloride, N,O‐bis(trimethylsilyl)acetamide, trimethylsilyl trifluoromethanesulfonate, magnesium sulfate, tetrabutylammonium fluoride [1.0 m in tetrahydrofuran (THF)], 4,4′‐dimethoxytrityl chloride, 3‐hydroxypropanenitrile, triethylamine, 2,4,6‐trimethylpyridine, and silver triflate were used as purchased from J&K (China). Activated carbon Darco G‐60, diisobutylaluminum hydride solution (1.0 m in toluene), acetic anhydride, sodium bicarbonate, sodium hydroxide, ammonium chloride, bis(diisopropylamino)chlorophosphine, acetic acid, urea, boric acid, ethylenediaminetetraacetic acid (EDTA) disodium salt dehydrate, formamide, magnesium chloride hexahydrate, StainsAll, tris(hydroxymethyl)aminomethane, (3‐aminopropyl) trimethoxysilane, *N*,*N*,*N*′,*N*′‐tetramethylethylenediamine, ammonium persulfate, glycerol, CelLytic M, and human serum from human male AB plasma were used as purchased from Sigma‐Aldrich. Acrylamide/bis‐acrylamide solution (19:1, 40%) was purchased from Bio‐Rad. A 1000 Å nucleoside‐derivatized long‐chain aminoalkyl‐controlled pore glass (CPG) solid support with loading densities of 25–40 µmol g^−1^, 1‐[(2‐cyanoethyl)‐(*N*,*N*‐diisopropyl)]‐phosphoramidite, and reagents used for automated DNA synthesis were purchased from BioAutomation. The EntrabsterTM, transfecting reagent was obtained from Engreen Biosystem Co, Ltd. (China).

### Instrumentation

Gel scanning was conducted using a Fujifilm FLA‐9000 scanner. Standard automated oligonucleotide solid‐phase synthesis was performed using a BioAutomation MerMade MM6 DNA synthesizer. Gel electrophoresis experiments were carried out using a 20 × 20 cm Maxi Vertical electrophoresis apparatus (MV‐20DSYS) with acrylamide. Confocal fluorescence imaging was performed using a laser confocal scanning microscope (Leica TCS SP5) with a magnification of 63 ×. The 3‐(4,5‐dimethylthiazol‐2‐yl)‐2,5‐diphenyltetrazolium bromide (MTT) experiment was conducted using a BioTek Powerwave XS microplate reader. Column chromatography utilized 60 Å 40–63 micron silica media (purchased from DAVISIL) as the solid support. Deuterated solvents were used as received from J&K. Fluorescence‐activated cell sorting studies were conducted using a BD FACSCantoTM II flow cytometer. The size of 3D spheroids was measured using NIS‐Elements D 5.02.03. The AnnexinV‐PE‐cy7 signal was detected using a Beckman Coulter CytoFLEX S Flow cytometer analyzer, utilizing the PC7 channel (780/60 BP).

### Synthesis and Characterization of TNA and DNA

Sequence‐designed TNA oligonucleotides were synthesized using an automatic nucleic acid synthesizer following our established solid‐phase synthetic protocols. Subsequently, the TNA strands were purified using the denaturing polyacrylamide gel electrophoresis (PAGE) method. The electrophoresis was carried out in 1 × TBE buffer at room temperature with a current of 30 mA for 1.5 h. To verify the successful synthesis of the sequence‐specified TNA polymers, MALDI‐TOF analysis was performed. Additionally, the quantification of TNA was determined through UV‐vis analysis.

### Cell Culture

MDA‐MB‐231, MDA‐MB‐468, BT‐549, HCC1806, and Hs578T TNBC cells were acquired from the American Type Culture Collection (ATCC, USA). MDA‐MB‐231/MDA‐MB‐468 cells were cultured in DMEM medium, while BT‐549 and HCC1806 cells were cultured in RPMI 1640 medium, Hs578T cells were cultured in the DMEM medium which contains one‐thousandth of the insulin content. Both culture media were supplemented with 10% FBS, penicillin (100 U mL^−1^), and streptomycin (100 µg mL^−1^). The cells were incubated at 37 °C in a humidified atmosphere with 5% CO_2_.

### Cytotoxicity Assay

The MDA‐MB‐231, MDA‐MB‐468, and BT‐549 cells in the logarithmic growth phase were subjected to trypsin digestion. The digestion process was halted by adding serum, and the cell suspension was centrifuged at 1500 rpm for 1 min, with the supernatant discarded. The cell pellet was resuspended to prepare a cell suspension at a concentration of 5 × 10^4^ cells mL^−1^. Once the cell suspension was prepared, it was gently mixed and seeded into a 96‐well plate. Each well was filled with 100 µL of the cell suspension, resulting in a seeding density of 10 000 cells per well. The edges around the experimental wells were sealed with PBS. Subsequently, the seeded cell culture plate was incubated overnight at 37 °C to ensure proper cell attachment to the plate. Next, different volumes of purified anti‐Akt TNAs were added to each well to achieve final concentrations of 0, 0.5, 1, 3, 5, and 10 µm within the culture plate, with 6 replicates for each concentration. The cell culture plate was further incubated at 37 °C for 48 h. After the incubation period, the culture media were removed, and 20 µL of MTT solution (5 mg mL^−1^) was added to each well, followed by a 4‐h incubation. The culture was then terminated, and the supernatant was aspirated. Subsequently, 150 µL of DMSO was added to each well and shaken at low speed on a shaker for 20 min to ensure complete dissolution of the crystals. Finally, the absorbance of each well at 570 nm was measured using a microplate reader.

### Fetal Bovine Serum Assay

The purified anti‐Akt TNAs (0.02 OD) were dissolved in 1 × PBS buffer supplemented with 10% Fetal Bovine Serum (FBS), resulting in a final concentration of 0.03 µg µL^−1^. Following that, the samples were incubated at 37 °C for various durations ranging from 0 to 72 h. A control group without the addition of FBS was established for comparison. After the designated incubation period, the samples were subjected to enzyme inactivation by incubating them at 60 °C for 20 min. Subsequently, the samples were analyzed using 15% PAGE. This process was conducted in 1 × TBE buffer at room temperature under a constant current of 30 mA. Finally, the gel was stained with stains‐All (50 mg L^−1^) and subsequently scanned for analysis.

### Confocal Fluorescence Microscopy Imaging

Typically, 1 × 10^5^ BT‐549 cells were seeded in a glass‐bottomed dish with a diameter of 35 mm and incubated overnight. The culture medium was then replaced with a medium containing 2.5 µg of anti‐Akt2 TNA‐Cy3, along with lipofectamine transfection reagents, and the cells were incubated for an additional 12 h. Subsequently, replaced the culture medium of the dishes followed by staining with a lysosome tracker for 10 min. Then, the cells were washed three times with PBS buffer at pH 7.4 and fixed with 4% paraformaldehyde for 15 min. After fixation, the cells were washed three times with 1 × PBS buffer at pH 7.4. Following the preparation of the fixed cells, confocal cell imaging experiments were conducted using a Leica TCS SP5 microscope. The excitation wavelength for Cy3 was set to 543 nm, and the emission was collected between 550 and 600 nm. The same steps were followed for the transfection of anti‐Akt3 TNA‐Cy3 into MDA‐MB‐231 cells.

### Quantitative Reverse Transcription Polymerase Chain Reaction

In this study, the concentration of scramble TNA and anti‐Akt TNA was fixed at 1.2 µg mL^−1^. Total RNAs were isolated from cells using the protocols provided in the RNeasy Mini Kit. For cDNA synthesis, 1 µg of total RNAs was used, and the TransScript First‐Strand Synthesis SuperMix Kit was employed. Real‐time amplification was conducted on a QuantStudio 3D Digital PCR system using the TransStart Tip Green qPCR SuperMix, following the manufacturer's instructions. PCR reactions (20 µL) were performed in triplicate under the following conditions: initial denaturation at 94 °C for 30 s, followed by 40 cycles of denaturation at 94 °C for 5 s, and annealing/extension at 60 °C for 30 s.

The primer sequences used for Akt2 were as follows:

Forward strand: 5′‐GCCGCCTTAATGCTAATCGTGAT‐3′

Reverse strand: 5′‐ATCCAGTGCAGGGTCCGAGG‐3′

The primer sequences used for Akt3 were as follows:

Forward strand: 5′‐CCGCTGCCTTGGACTATCTACA‐3′

Reverse strand: 5′‐TGCGATCGCCAGTTCCGTAA‐3′

Glyceraldehyde‐3‐phosphate dehydrogenase (GAPDH) was utilized as an internal control for normalization. Data analysis was performed using the Quant Studio Design and Analysis Software.

### Western Blot

In Western blot analysis, the procedure begins with the preparation of separating and stacking gels based on the target protein's molecular weights. After solidifying the 4.5 ml of separating gel for ≈30 min, 1.5 mL of the stacking gel was added, and the gel was left to set. Following this, the gel comb was removed, and each well was rinsed. 15–30 µg of MDA‐MB‐231 or BT‐549 cell proteins were added to the wells, and gel electrophoresis at a voltage of 80V in 1 × electrophoresis buffer was carried out for ≈2.5 h. Once electrophoresis was almost complete, a PVDF membrane was prepared and activated using methanol and transfer buffer. The gel was then cut according to the desired protein weights and transferred onto the membrane using a voltage of 100 V for 3 h. Subsequently, the membrane was washed and blocked with bovine serum albumin. Incubation with diluted primary antibodies for Akt2 or Akt3 was performed overnight at 4 °C, followed by washing and incubation with secondary antibodies at room temperature. After additional washing steps, the protein bands on the membrane were visualized using detection methods. Finally, the obtained results were analyzed to determine the presence and relative abundance of Akt2 and Akt3 proteins.

### 3D Multicellular Spheroid Preparation

To initiate the experiment, agarose‐coated 48‐well plates were prepared. First, a 1% agarose solution in PBS buffer was prepared and heated in a microwave oven until fully dissolved. Next, 0.3 mL of the heated agarose solution was dispensed into each well of the plates. The plates were then allowed to cool and solidify, ensuring proper agarose coating. After that, MDA‐MB‐231 cells were seeded in the agarose‐coated 48‐well plates at a density of 5000 cells per well. The plates were placed on a shaker at 50 rpm for 24 h to facilitate the formation of multicellular spheroids. Following this, the cells were incubated in a cell incubator, with medium renewal every 2 days, until the multicellular spheroids reached a diameter of over 500 µm. To obtain multicellular spheroids with varying diameters, different cell densities were used for seeding MDA‐MB‐231 cells in agarose‐coated 48‐well plates. Additionally, BT‐549 multicellular spheroids were also prepared to compare their morphology with the MDA‐MB‐231 spheroids. Throughout the process, utmost care was taken to prevent the disintegration of the multicellular spheroids.

### 3D Cell Growth Assay

The 3D cultures were prepared following the previously described method.^[^ ^49^ ^]^ Briefly, 96‐well plates (Corning #3610) were coated with growth factor‐reduced Matrigel (BD Biosciences). A total of 1000–1300 cells were seeded in assay medium, which consisted of DMEM or RPMI 1640 supplemented with 10% FBS and 2% Matrigel. The assay medium was refreshed every 2 days, and on day 4 and day 6, 172 ng of TNA was added. On day 8, images were captured using a Nikon Ts2 FL inverted microscope with 4× and 10× objectives.

### 3D Annexin V Apoptosis Detection assay

The Annexin V Apoptosis Detection Kit (eBioscience, #88‐8103‐72) was utilized to detect cell apoptosis through the use of fluorescently labeled annexin V. After transfection, 20 × 10^4^ cells were seeded in each well of a homemade ultra‐low attachment 6‐well plate in assay medium. The assay medium was refreshed every 2 days, and on day 4 and day 6, 2.75 µg of TNA was added. On day 8, the spheroids were collected and dissociated into single cells. Subsequently, 20 × 10^4^ cells were incubated with AnnexinV‐PE‐cy7 in a binding buffer for 15 min at room temperature, protected from light. The cells were then centrifuged, and the pellets were resuspended with binding buffer on ice. The AnnexinV‐PE‐cy7 signal was detected using the PC7 channel (780/60 BP) of the Beckman Coulter CytoFLEX S flow cytometer analyzer.

### Animal Studies

The BALB/c nude mice (6–8 weeks) were purchased from the Laboratory Animal Research Unit (LARU) of City University of Hong Kong and housed in a pathogen‐free environment. All animal experiments were approved by the Animal Ethics Committee of City University of Hong Kong and carried out following the Guidelines for Care and Use of Laboratory Animals of City University of Hong Kong.

Cells in logarithmic growth phase, with a count of 5 × 10^6^ BT‐549 or MDA‐MB‐231 cells, were harvested, digested, and combined with matrix gel in a 1:1 ratio. The resulting mixture was kept on crushed ice and prepared for inoculation into nude mice at the animal facility. A 100 µL aliquot of the cell suspension was extracted and injected between the skin and muscle of the mammary gland, resulting in the formation of a smooth, round bump, indicating successful model construction. When the tumor volume reached ≈100 mm^3^ (set as day 1), the mice were randomly assigned to three groups with *n* = 4 for in vivo antisense cancer therapy: 1) PBS group; 2) Scramble TNA group; 3) anti‐Akt TNA group.

### Preparation of Anti‐Akt TNAs/LNP for In Vivo Injection

First, 50 µg of Anti‐Akt2 TNA was dissolved in 50 µL of water, resulting in a final concentration of 1 mg mL^−1^. This solution was then mixed with a 10% glucose solution (w/v) to achieve a final glucose concentration of 5% in a total volume of 100 µL. Second, 25 µL of EntransterTM‐in vivo reagent was mixed with 50 µL of 10% glucose solution, followed by the addition of 25 µL of pure water to obtain a final glucose concentration of 5% in a total volume of 100 µL. Subsequently, these two solutions were thoroughly mixed and immediately injected into the tumor site as a single dose, with a total volume of 200 µL for the test complex. This injection was repeated every two days for a total of four times. Changes in tumor size and body weight were then observed and recorded in the mice for a duration of 21 days.

### Immunohistochemistry (IHC)

The tissue sections undergo a series of steps to prepare them for analysis. Initially, the sections were deparaffinized by dehydrating them using a range of ethanol concentrations, followed by absolute ethanol, and then transferring them through xylene solutions. Subsequently, the sections were immersed in soft and hard paraffin for embedding. After embedding, the tissue blocks were chilled to facilitate easier sectioning. The blocks were trimmed to expose the tissue, and sections with a thickness of 4–7 µm were cut. To prepare the sections for staining, another deparaffinization process was performed using xylene and ethanol solutions, followed by rinsing with water. Immunohistochemistry was then conducted on the tissue sections. The sections were treated with H_2_O_2_ to eliminate endogenous peroxidase activity and undergo rinsing steps. Antigen retrieval was performed using a citrate buffer, followed by rinsing with PBS. A blocking solution was applied, and the sections were incubated. After discarding the blocking solution, the sections were incubated with the primary antibody, followed by incubation with the secondary antibody. Rinsing with PBS was carried out between antibody incubations. To visualize the target molecules, DAB staining was performed, and the staining process was halted once brownish‐yellow particles appeared. The sections were rinsed and counterstained with hematoxylin. Dehydration was achieved using xylene and ethanol solutions, and finally, the sections were mounted using a neutral mounting medium.

### Statistical Analysis

The data presented in this study were derived from a minimum of three independent experiments. All results were reported as the mean ± standard deviation (SD). Statistical analysis was performed using Prism and Origin software, and comparisons among multiple groups were evaluated using one‐way analysis of variance (one‐way ANOVA). Statistical significance was determined as follows: Error bars represent the mean ± standard error of the mean (SEM) from three independent experiments. *p < 0.05, **p < 0.01, ***p < 0.001, ****p < 0.0001. The p‐values were calculated using a two‐sided Student's t‐test.

## Conflict of Interest

The authors declare that they have no conflict of interest.

## Supporting information

Supporting Information

## Data Availability

The data that support the findings of this study are available in the supplementary material of this article.
