# Supplementary material for: TNA‐Mediated Antisense Strategy to Knockdown Akt Genes for Triple‐Negative Breast Cancer Therapy
Source: Small Methods. 2024 May 23;8(11):2400291. doi: 10.1002/smtd.202400291 (PMC11579567; doi:10.1002/smtd.202400291)
Supplement: Supplementary file 1 — Supporting Information [file SMTD-8-2400291-s001.pdf]

small
methods

Supporting Information

for *Small Methods*, DOI 10.1002/smtd.202400291

TNA-Mediated Antisense Strategy to Knockdown Akt Genes for Triple-Negative Breast Cancer Therapy

Pan Li, Shixue Zheng, Hoi Man Leung, Ling Sum Liu, Tristan Juin Han Chang, Alishba Maryam, Fei Wang, Y. Rebecca Chin* and Pik Kwan Lo**

Supporting Information

TNA-Mediated Antisense Strategy To Knockdown AKT Genes For Triple-Negative Breast Cancer Therapy

Pan Li[a], Shixue Zheng[b], Hoi Man Leung[a], Ling Sum Liu[c], Tristan Juin Han Chang[a], Alishba Maryam[b], Fei Wang[d] *, Y. Rebecca Chin [b] *, Pik Kwan Lo[a,e] *

a. Department of Chemistry and State Key Laboratory of Marine Pollution, City University of Hong Kong, Tat Chee Avenue, Kowloon, Hong Kong SAR, P. R. China
b. Tung Biomedical Sciences Centre, Department of Biomedical Sciences, City University of Hong Kong, Kowloon, Hong Kong, SAR, P. R. China
c. Department of Chemistry, Molecular Sciences Research Hub, Imperial College London, White City Campus, Wood Lane, London W12 0BZ, United Kingdom
d. The Tenth Affiliated Hospital, Southern Medical University (Dongguan People's Hospital), 523059, Dongguan, P. R. China
e. Key Laboratory of Biochip Technology, Biotech and Health Care, Shenzhen Research Institute of City University of Hong Kong, 518057, Shenzhen, P. R. China

Corresponding E-mail: peggylo@cityu.edu.hk

(a)

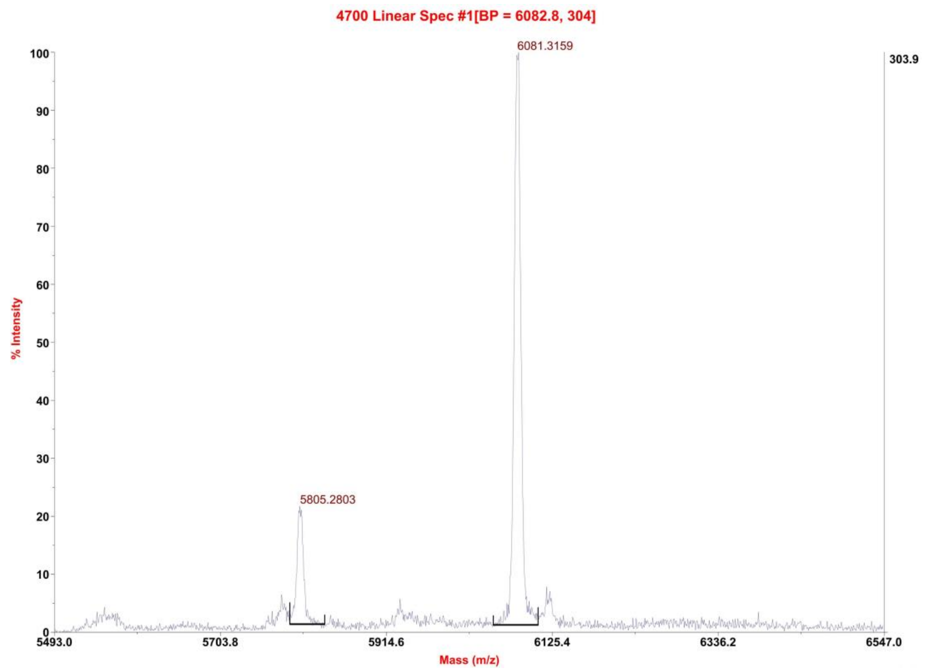
4700 Linear Spec #1[BP = 6082.8, 304]
6081.3159
5805.2803
303.9
% Intensity
Mass (m/z)
5493.0
5703.8
5914.6
6125.4
6336.2
6547.0

(b)

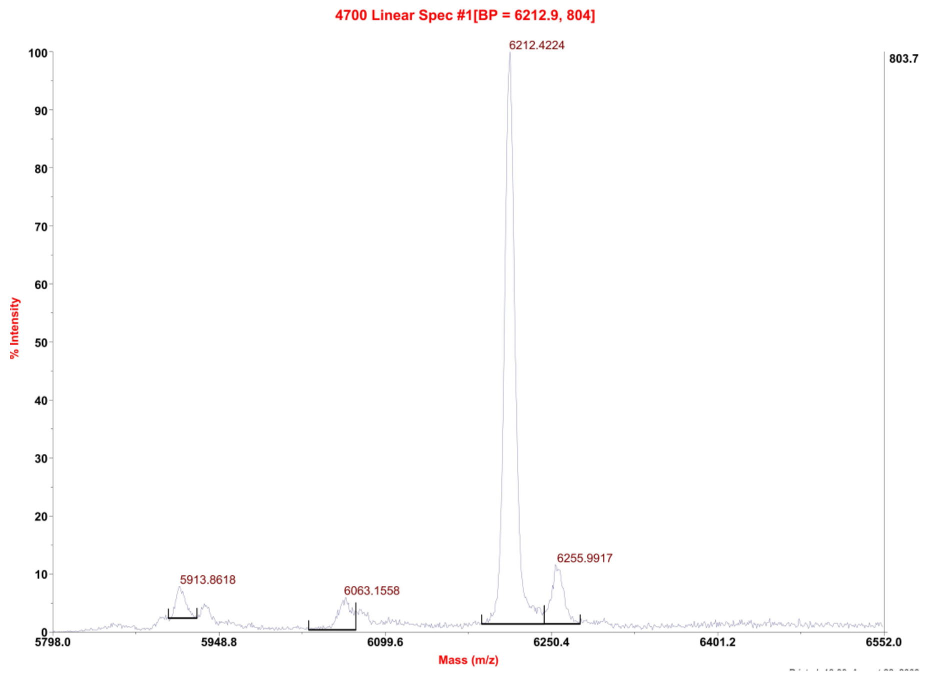
4700 Linear Spec #1[BP = 6212.9, 804]
6212.4224
6255.9917
5913.8618
6063.1558
803.7
% Intensity
Mass (m/z)
5798.0
5948.8
6099.6
6250.4
6401.2
6552.0

(c)

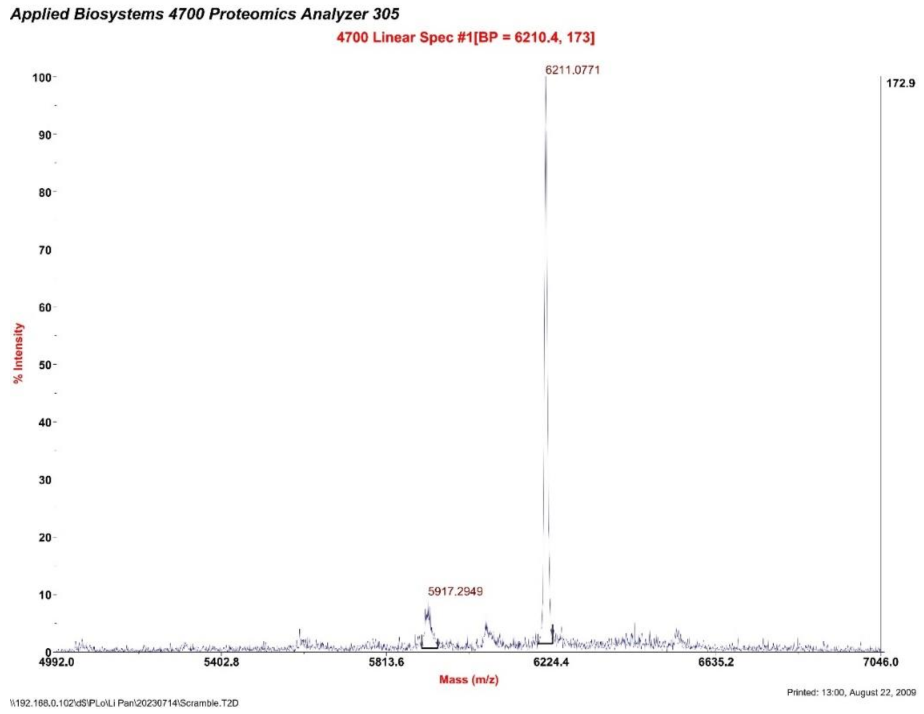
Applied Biosystems 4700 Proteomics Analyzer 305
4700 Linear Spec #1[BP = 6210.4, 173]
6211.0771
172.9
5917.2949
% Intensity
Mass (m/z)
Printed: 13:00, August 22, 2009
\\192.168.0.102\d$\PLo\Li Pan\20230714\Scramble.T2D

(d)

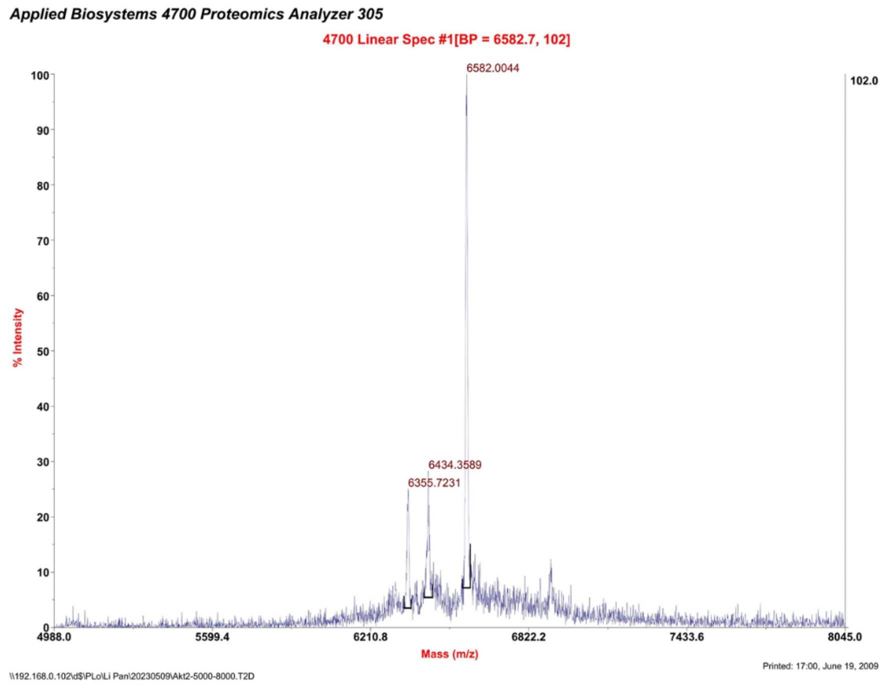
Applied Biosystems 4700 Proteomics Analyzer 305
4700 Linear Spec #1[BP = 6582.7, 102]
6582.0044
102.0
6434.3589
6355.7231
% Intensity
Mass (m/z)
Printed: 17:00, June 19, 2009
\\192.168.0.102\d$\PLo\Li Pan\20230509\Akt2-5000-8000.T2D

(e)

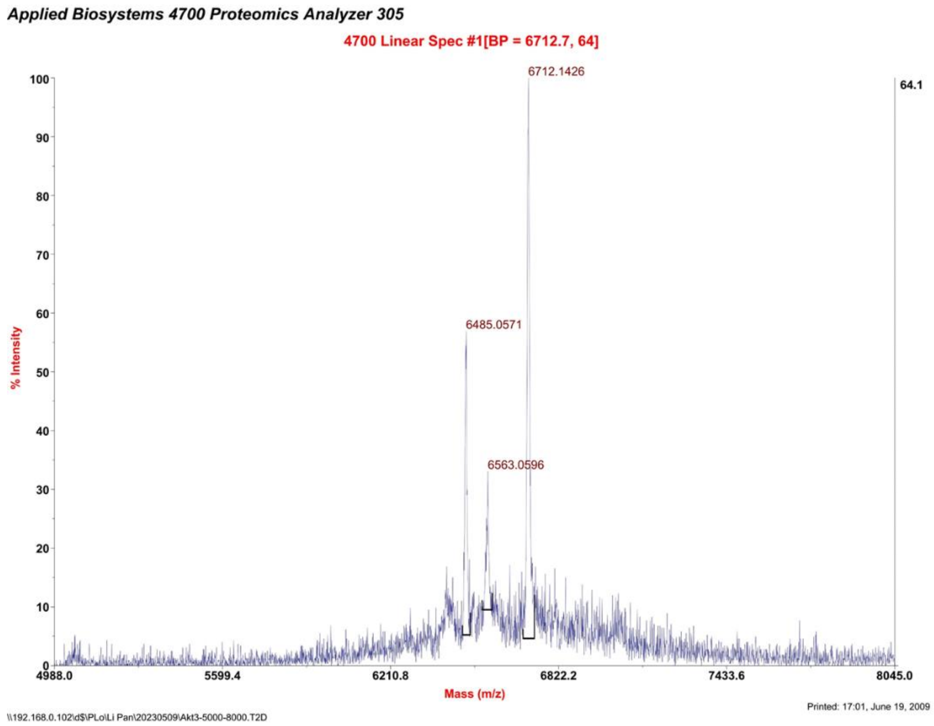

Figure S1. (a) MALDI-TOF spectrum of anti-Akt2 TNA of m/z calculated 6136.4353; found 6081.3159 [M^+]; (b) anti-Akt3 TNA of m/z calculated 6267.5653; found 6212.4224 [M^+]; (c) scramble TNA of m/z calculated 6267.5653; found 6211.0771 [M^+]; (d) Anti-Akt2 TNA-Cy3 of m/z calculated 6598.23; found 6582.004 [M^+]; (e) and Anti-Akt3 TNA-Cy3 of m/z calculated 6725.37; found 6712.143 [M^+].

Table S1. Sequences related in this project

Type	Name of oligonucleotide	Sequence	Synthetic yield
**RNA**	Sense-Akt2 RNA	5’-GCGUGGUGAAUACAUCAAGAC-3’	-
	Sense-Akt3 RNA	5’-CUGCCUUGGACUAUCUACAUU-3’	-
**DNA**	Sense-Akt2 DNA	5’-GCGTGGTGAATACATCAAGAC-3’	-
	Sense-Akt3 DNA	5’-CTGCCTTGGACTATCTACATT-3’	-

**TNA**	Anti-Akt2 TNA	3’-GTCTTGATGTATTCACCACGC-2’	537.9 μg (73.0%)
	Anti-Akt2 TNA-Cy3	3’-GTCTTGATGTATTCACCACGC-Cy3-2’	468.6 μg (63.3%)
	Anti-Akt3 TNA	3’-AATGTAGATAGTCCAAGGCAG-2’	382.9 μg (51.8%)
	Anti-Akt3 TNA-Cy3	3’-AATGTAGATAGTCCAAGGCAG-Cy3-2’	488.4 μg (66.1%)
	Scramble TNA	3’-TAGGATCTACACAGAGATGAG-2’	580.8 μg (78.5%)

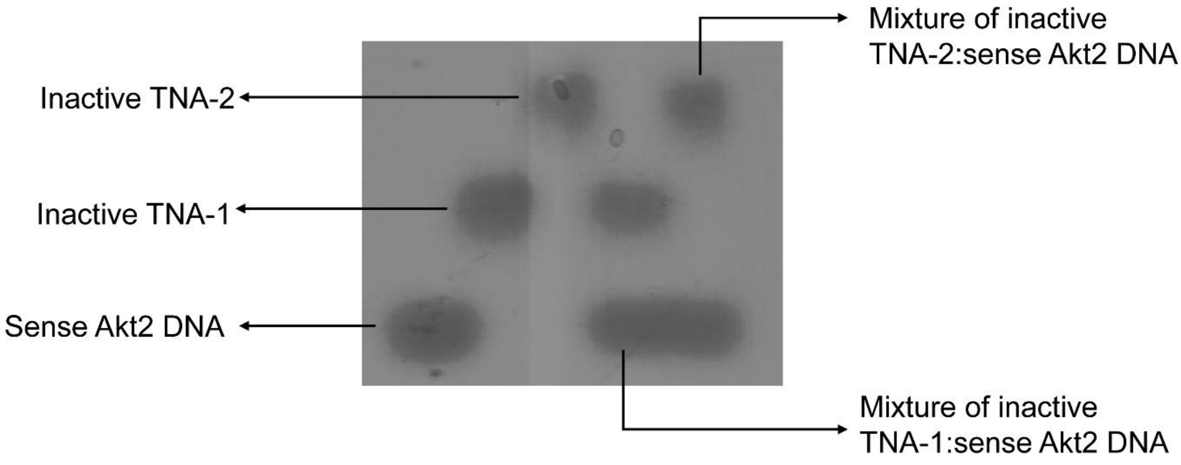

Figure S2. Native PAGE analyses of the binding property of the anti-Akt2 TNA and anti-Akt3 TNA with non-complementary DNA and/or RNA.

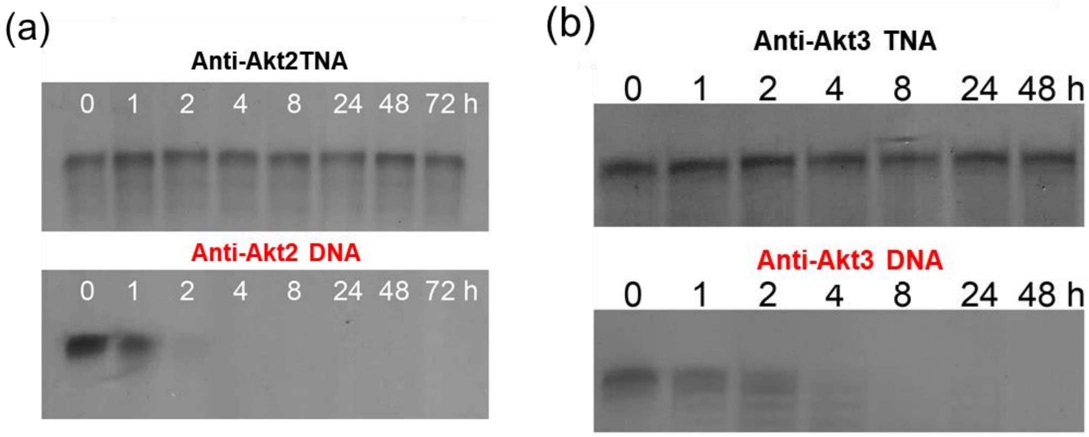

Figure S3. Denaturing PAGE analysis of (a) anti-Akt2 TNA/DNA and (b) anti-Akt3 TNA/DNA, after incubation with 10 % FBS for the designed time intervals.

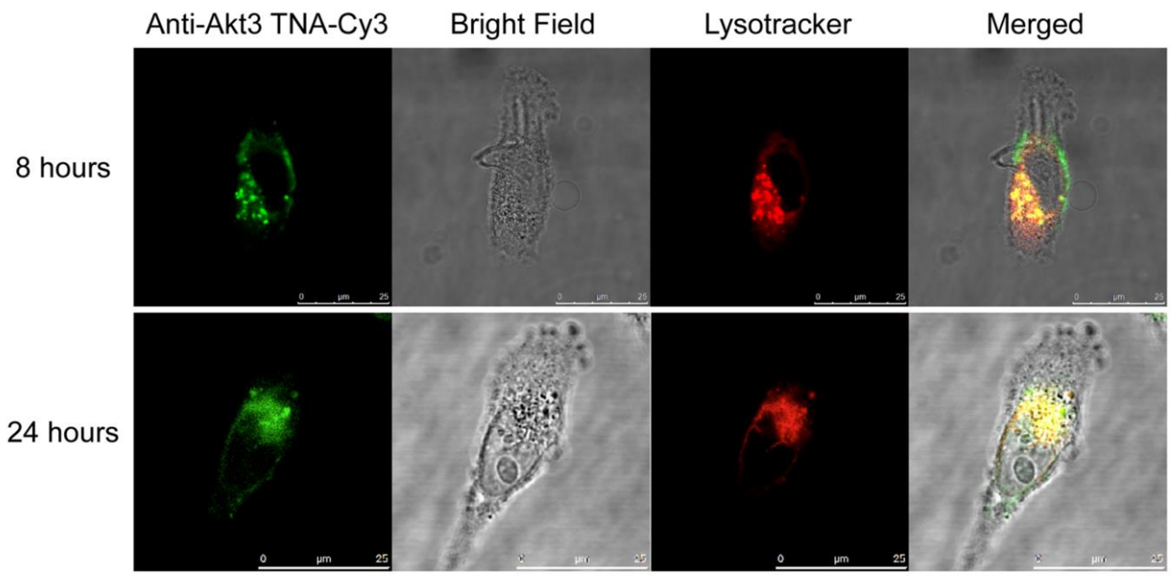

Figure S4. Confocal fluorescence images of anti-Akt3 TNA-treated MDA-MB-231 cells after incubating of 8 h and 24 h. Scale bar = 50 µm.

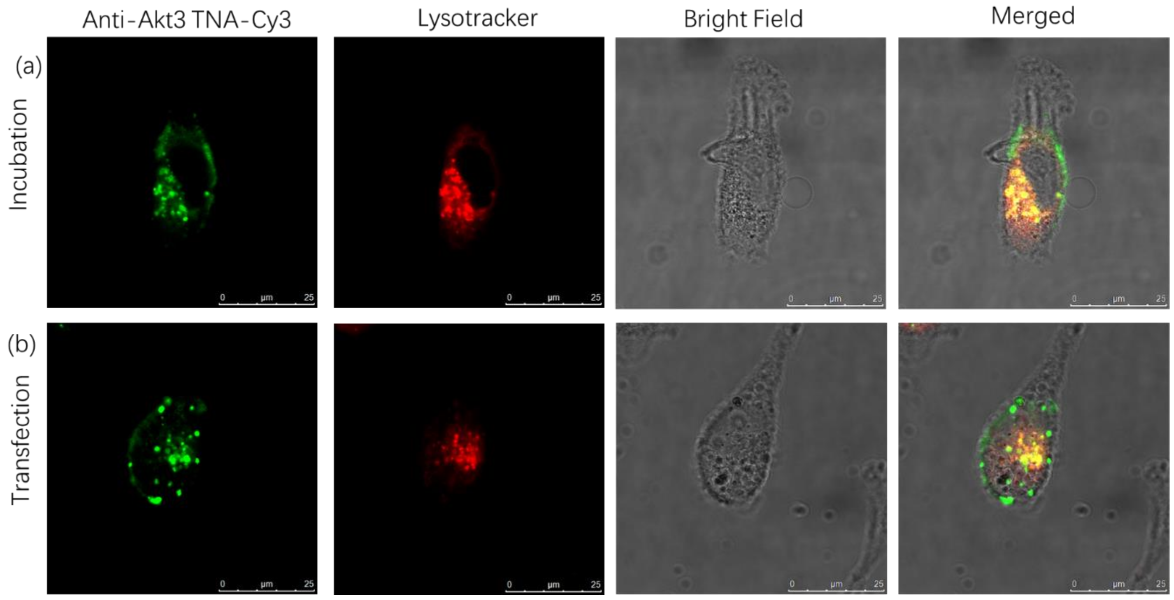

Figure S5. Confocal fluorescence images of anti-Akt3 TNA-treated MDA-MB-231 cells (a) with and (b) without using lipofectamine-transfecting agent after incubating of 8 h. Scale bar = 50 µm.

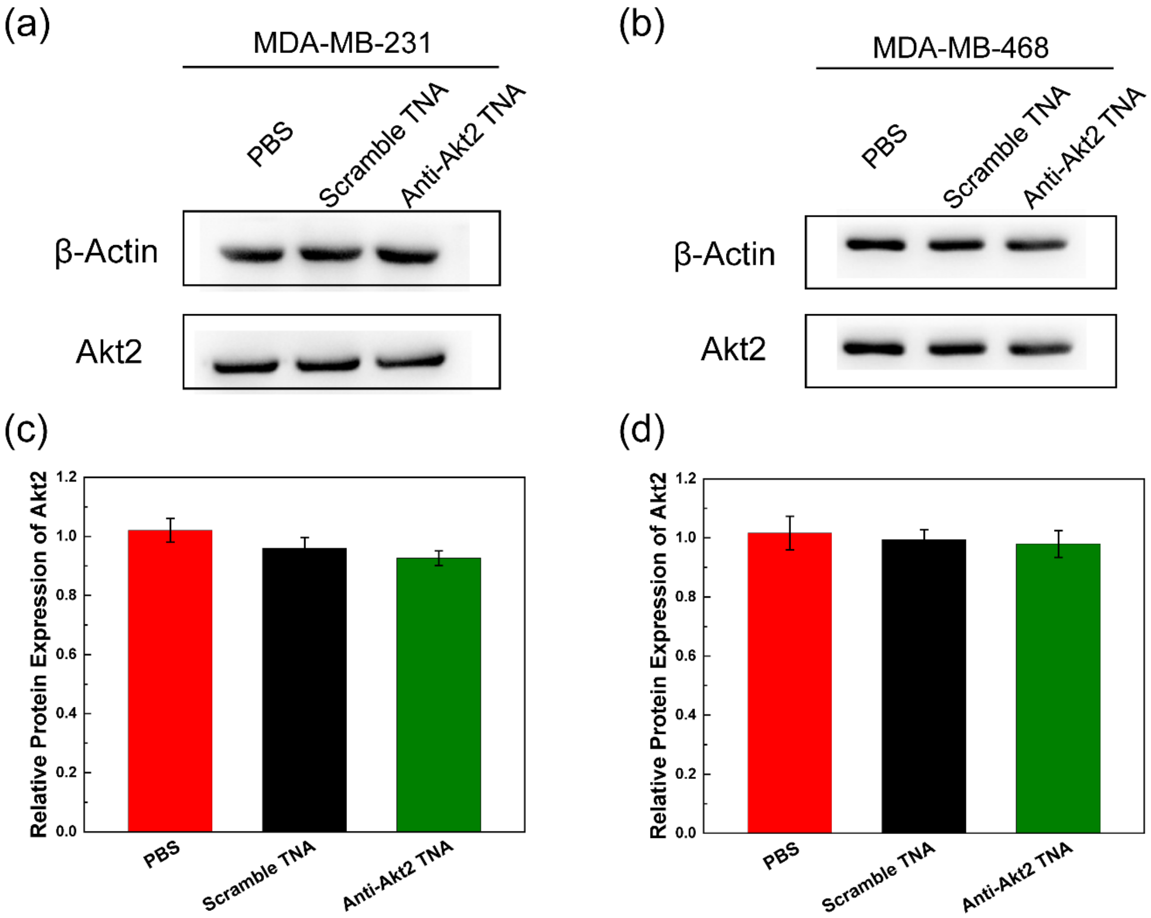

Figure S6. Akt2 protein expression level of (a) MDA-MB-231 and (b) MDA-MB-468 cells after different treatments. Quantitative analyses of Akt2 protein in (c) MDA-MB-231 cells and (d) MDA-MB-468 cells in (a) and (b) respectively.

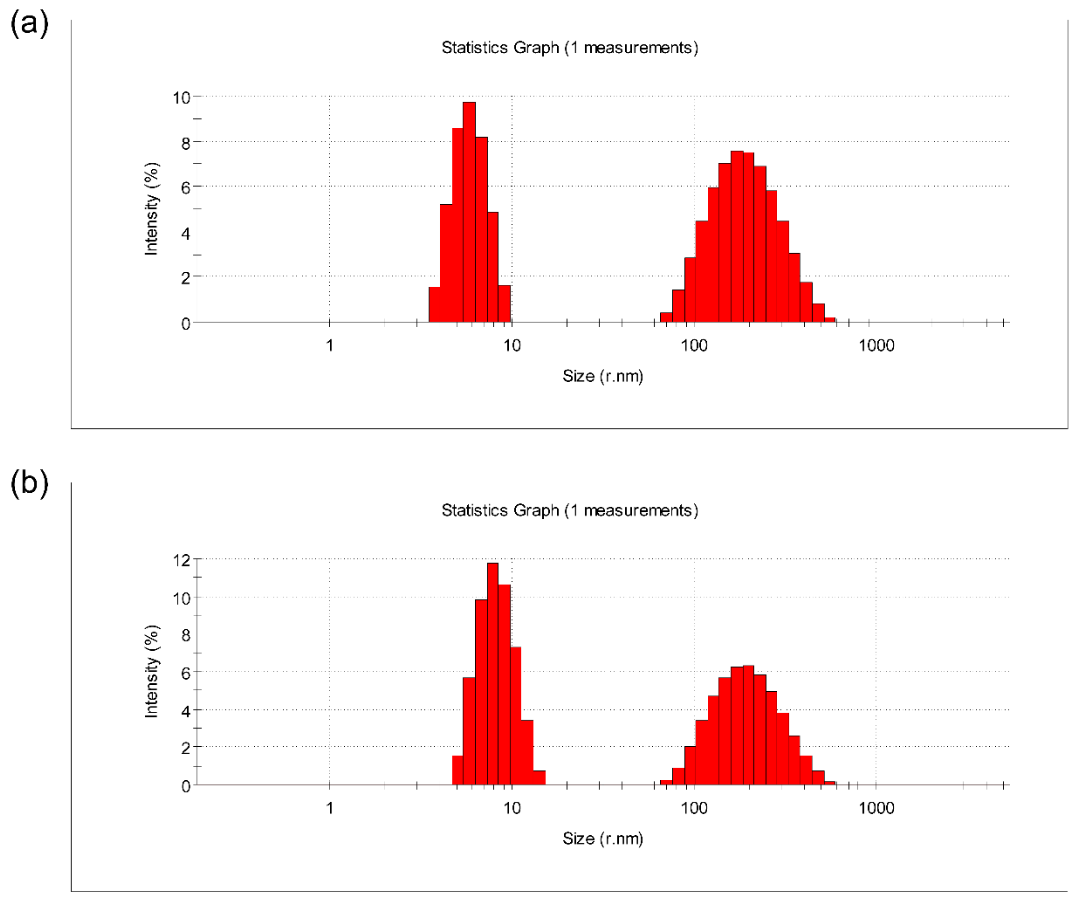

Figure S7. The DLS results of LNP measured on (a) day 1 and (b) day 3 at the concentration of 10 μg/mL.

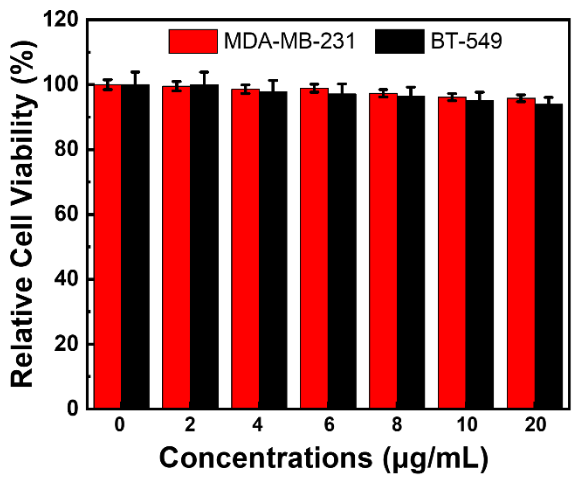

Figure S8 The cell viability of LNP-treated BT549 and MDA-MB-231 cells as a function of concentration.
